# A case of unilateral lung collapse

**DOI:** 10.11604/pamj.2023.46.43.41281

**Published:** 2023-09-29

**Authors:** Ashwin Karnan, Ulhas Jadhav

**Affiliations:** 1 Department of Respiratory Medicine, Jawaharlal Nehru Medical College, Datta Meghe Institute of Higher Education and Research, Sawangi (Meghe), Wardha, Maharashtra, India

**Keywords:** Mucus, cough, breathlessness, lung collapse, atelectasis

## Image in medicine

A 10-month-old infant was brought to our emergency department with complaints of acute respiratory distress for the past half an hour with no significant family, birth, or history. On examination, pulse rate (PR) was 130 beats/min, saturation 75% on room air, respiratory rate (RR) of 32 breaths/min, blood pressure of 100/70mmhg, and on auscultation, there were absent breath sounds on the right side. Chest X-ray showed opaque right hemithorax. An emergency bronchoscopy was done, which showed thick mucus plugs in the right segmental bronchi causing lung collapse. Mucus plugs were removed and post-procedure Chest X-ray showed complete resolution of the right lung. The mucus is a mixture of water, proteins, proteoglycans, lipids, and secretions from the goblet cells, which line most of the respiratory tract. This mucus along with the continuous movement of cilia on respiratory epithelium helps to prevent dust particles, pollutants, and infectious agents from entering the system. In conditions where there is delayed mucociliary clearance or mucus hypersecretion, this mucus accumulation may cause cough and narrowing of airways resulting in a decline in lung function. In bronchial asthma, allergic bronchopulmonary aspergillosis, cystic fibrosis, and primary ciliary dyskinesia there may be mucus accumulation which may lead to bacterial colonization. Decongestants, mucolytics, coughing techniques, and chest physiotherapy in the form of chest percussion and postural drainage may aid in reducing secretions.

**Figure 1:**
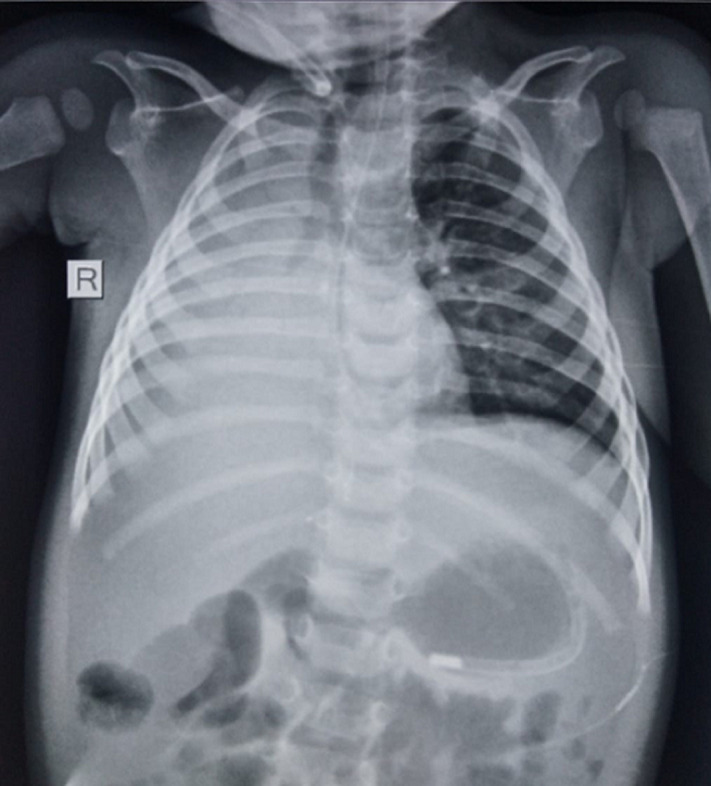
chest X-ray showing right opaque hemithorax with trachea pulled to the right side with nasogastric tube in situ

